# Plasma-Derived Exosomal Circular RNA hsa_circ_0005540 as a Novel Diagnostic Biomarker for Coronary Artery Disease

**DOI:** 10.1155/2020/3178642

**Published:** 2020-06-30

**Authors:** Wei-peng Wu, Yan-hong Pan, Meng-yun Cai, Jin-ming Cen, Can Chen, Lei Zheng, Xinguang Liu, Xing-dong Xiong

**Affiliations:** ^1^Guangdong Provincial Key Laboratory of Medical Molecular Diagnostics, Institute of Aging Research, Guangdong Medical University, Dongguan 523808, China; ^2^Institute of Biochemistry & Molecular Biology, Guangdong Medical University, Zhanjiang 524023, China; ^3^Department of Cardiovascular Disease, The First People's Hospital of Foshan, Foshan 528000, China; ^4^Department of Cardiovascular Disease, The Affiliated Hospital of Guangdong Medical University, Zhanjiang 524023, China; ^5^Department of Laboratory Medicine, Nanfang Hospital, Southern Medical University, Guangzhou 510515, China

## Abstract

**Background:**

Exosomes exist in almost all body fluid and contain diverse biological contents which may be reflective of disease state. Circular RNAs (circRNAs) are stable in structure and have a long half-life in exosomes without degradation, thus making them reliable biomarkers. However, the potential of exosomal circRNAs as biomarkers of coronary artery disease (CAD) remains to be established. Here, we aimed to investigate the expression levels and the potential use of exosomal circRNAs as diagnostic biomarkers for CAD.

**Methods:**

CircRNA expression levels in exosomes obtained from three plasma samples of CAD patients and three paired controls were analyzed using RNA sequencing. Exosomal circRNAs obtained in the profiling phase were then verified in two-center validation cohorts. Finally, the ability of exosomal circRNAs, adjusting for Framingham Heart Study (FHS) risk factors, was determined to discriminate between CAD patients and non-CAD controls.

**Results:**

355 circRNAs were differentially expressed between these two groups: 164 were upregulated, and 191 were downregulated. Here, we selected the potential circRNAs (fold change > 4, *P* < 0.05) as candidate biomarkers for further validation. Our data showed that only hsa_circ_0005540 was significantly associated with CAD (*P* < 0.0001). After adjustment for risk factors, hsa_circ_0005540 showed a high discriminatory power for CAD in ROC analyses (AUC = 0.853; 95%confidence interval (CI) = 0.799 − 0.906, *P* < 0.001).

**Conclusion:**

Our results suggest that plasma exosomal hsa_circ_0005540 can be used as a promising diagnostic biomarker of CAD.

## 1. Introduction

CAD is one of the most common forms of cardiovascular diseases and remains a leading cause of mortality worldwide. It poses a high economic burden to the national healthcare system mainly due to its late diagnosis and severe complications [1, 2]. Among the current clinical diagnosis methods, invasive coronary angiography (CAG) is the diagnostic “gold standard,” but its specialized technology and high cost limit it to a selective population [3]. Although the current standardized treatment strategies for CAD, which include coronary bypass surgery, balloon dilatation of coronary vessels, and percutaneous transluminal coronary angioplasty, have been developed, the survival of the patients has remained dismal and the prognosis ineligible [4, 5]. The main reason accounting for this discrepancy is mainly due to the current diagnostic methods unable to simultaneously achieve high sensitivity and convenience. Thus, highly sensitive and convenient diagnostic biomarkers are urgently needed for diagnosis of CAD.

Exosomes are a class of nanoscale (30-150 nm) extracellular vesicles that can be generated and released by most cell types [6]. They are generated within the endosomal compartment and released into the extracellular environment after the fusion of multivesicular bodies (MVBs) with the plasma membrane [7–9]. These vesicles have been proved to be accessible in nearly all body fluids such as the blood [10], urine [11, 12], and saliva [13]. Exosomes can transfer functional RNAs, proteins, and lipids to adjacent cells and serve as mediators of intercellular communication [14–16]. In addition, exosomes have a relatively stable structure which can protect their cargos from destruction, thus making them potential candidates for noninvasive biomarkers [17].

CircRNAs are a large novel type of endogenous transcripts and represent a recent research hotspot in the field of RNA. They form covalently closed loop structures with neither 5′-3′ polarities nor a polyadenylated tail, which makes them much more stable than linear RNAs [18–20]. Recent studies showed that circRNAs could function as miRNA sponges, bind partners of proteins, regulators of alternative splicing, or even be translated into proteins driven by N6-methyladenosine modification [21–25]. Additionally, high abundance, relative stability, tissue/developmental-stage-specific expression, and evolutionary conservation among species make circRNAs very attractive for clinical application [18, 26, 27]. Due to those characteristics, circRNAs have a great potential to serve as significant biomarkers to diagnose diseases.

Recent studies confirmed that circRNAs are enriched and stable in exosomes and can continually play their roles after exosomes are secreted from the parent cells into the bodily fluid [28, 29]. There are growing evidences of exosomal circRNA biomarkers for various cancers [29–31]. For instance, it was reported that serum exosomal circKLDHC10 could distinguish patients with colorectal cancer from healthy controls, suggesting its significant potential as a circulating biomarker for cancer diagnosis [29]. However, the functional roles of exosomal circRNAs in CAD remain elusive. Thus, the current study is aimed at assessing the differential expressions of exosomal circRNAs in the plasma of CAD patients and non-CAD controls and to determine their potential diagnostic value for CAD.

## 2. Materials and Methods

### 2.1. Study Subjects

Sixty-one CAD patients and thirty-eight non-CAD controls enrolled from center 1 (the First People's Hospital of Foshan) between March 2018 and August 2018 formed the profiling and internal validation phases. Forty-seven CAD patients and 51 non-CAD controls recruited at center 2 (the Affiliated Hospital of Guangdong Medical University) between January 2019 and May 2019 constituted the external validation phase. The diagnosis of CAD was based on the percentage narrowing of each coronary artery segment, with 50% stenosis of at least one of the major coronaries (the left main coronary trunk, anterior descending branch, circumflex artery, and right coronary artery). Patients with impaired ejection fraction, heart failure, or acute myocardial injury were excluded. Age-, sex-, and ethnicity-matched subjects were used as non-CAD controls. All the controls have normal electrocardiogram (ECG) records and no evidence of CAD. General exclusion criteria were a known history of leukopenia, thrombocytopenia, or severe hepatic or renal dysfunction, as well as evidence for inflammatory or malignant disease. Written informed consent was obtained from each of the participants. This study was approved by the Ethics Committee of the First People's Hospital of Foshan and the Affiliated Hospital of Guangdong Medical University under the guidance of the Helsinki Declaration. The characteristics of the study subjects are shown in Table 1.

### 2.2. Exosomal RNA Isolation

For exosomal RNA extraction, an exoRNeasy Serum/Plasma Midi kit (Qiagen, CA, USA) was used to isolate total exosome RNA from 1 ml prefiltered plasma for validation cohorts according to the manufacturer's protocol. Briefly, prefiltered plasma was mixed 1 : 1 with 2x binding buffer (XBP) and added to the exoEasy membrane affinity column to bind the exosomes to the membrane. After centrifugation, the flow-through was discarded and the wash buffer (XWP) was added to the column to wash off nonspecifically retained material. After another centrifugation and discarding of the flow-through, the plasma exosomes were lysed by adding QIAzol to the spin column, and the lysate was collected by centrifugation. Following the addition of chloroform, thorough mixing and centrifugation to separate organic and aqueous phases, the aqueous phase was recovered and mixed with ethanol. The sample-ethanol mixture was added to the RNeasy MinElute spin column and centrifuged. The column was washed once with buffer RWT, and then twice with buffer RPE followed by elution of RNA in water. Collected RNA was then used in the subsequent experiments.

### 2.3. Exosomal CircRNA Sequence Analysis

Total RNA for RNA-seq analysis was extracted using an exoRNeasy Serum/Plasma Maxi kit (Qiagen, CA, USA) from 4 ml prefiltered plasma according to the manufacturer's protocol. The purity and concentration of RNA were determined from the OD260/280 readings using a spectrophotometer (NanoDrop ND-1000). The RNA integrity was assessed using Agilent Bioanalyzer 2100 (Agilent Technologies). Total RNA from each sample was subjected to the RiboMinus Eukaryote Kit (Qiagen, CA, USA) to remove ribosomal RNA before the construction of RNA-seq libraries. Strand-specific RNA-seq libraries were prepared using NEBNext Ultra Directional RNA Library Prep Kit for Illumina (NEB) following the manufacturer's instructions. Briefly, approximately 50 ng of ribosome-depleted RNA samples were fragmented and then used for first- and second-strand cDNA syntheses with random hexamer primers. For second-strand cDNA synthesis, dUTP mix (without dTTP) was used which allows the removal of the second strand. An End-It DNA End Repair Kit was used to repair the ends of the double-stranded cDNA fragments, which were then modified by the Klenow fragment so that an A was added to the 3′ end of the DNA fragments; the fragments were finally ligated to adapters. The ligated cDNA products were purified and treated with uracil DNA glycosylase to remove the second-strand cDNA. Purified first-strand cDNA was subjected to 14 cycles of PCR amplification, followed by library analysis with a Bioanalyser 2100 (Agilent, CA, USA). The strand-specific RNA-seq libraries were sequenced using Illumina HiSeq™ 2500 platform.

### 2.4. Quantitative Real-Time RT-PCR

Total RNAs were converted to cDNA using PrimeScript™ RT Reagent Kit (Takara, Otsu, Japan). The differentially expressed exosomal circRNAs selected from the profiling data were measured by quantitative real-time RT-PCR using the LightCycler® 96 detection system at 95°C for 3 min and amplified by 40 cycles of denaturing at 95°C for 10 s and 60°C for 30 s. The CT value was the fractional cycle number at which the fluorescence exceeded the given threshold. *ACTIN* was used to normalize the RNA preparation. The relative fold-change was calculated using the 2^-*ΔΔ*CT^ method. Primer sequences are listed in TABLE S1 (see TABLE S1 in the Supplementary Material).

### 2.5. Statistical Analysis

The differences of the demographic, clinical pathological characteristics and circRNA expression levels between the CAD patients and non-CAD controls were estimated using Student's *t*-test and *χ*2 test for continuous variables and categorical variables, respectively. Data were expressed as percentages for the qualitative variables and mean ± standard deviation (SD) for the quantitative variables. In the scatter plot depicting the circRNA expression, the horizontal lines represent the medians. The area under the receiver operator characteristic (ROC) curve (AUC) was used to evaluate the diagnostic value of exosomal circRNAs for CAD. The corresponding sensitivity and specificity were identified through the ROC curve analysis. Multivariate logistic regression models were constructed with stepwise addition of FHS risk factors (age, gender, smoking history, diabetes, hypertension, and TC and HDL levels) for each circRNA. All statistical analyses were performed using SPSS software. *P* < 0.05 was considered statistically significant.

## 3. Results

### 3.1. Profiling of Exosomal CircRNAs in CAD Patients

**Figure 1:**
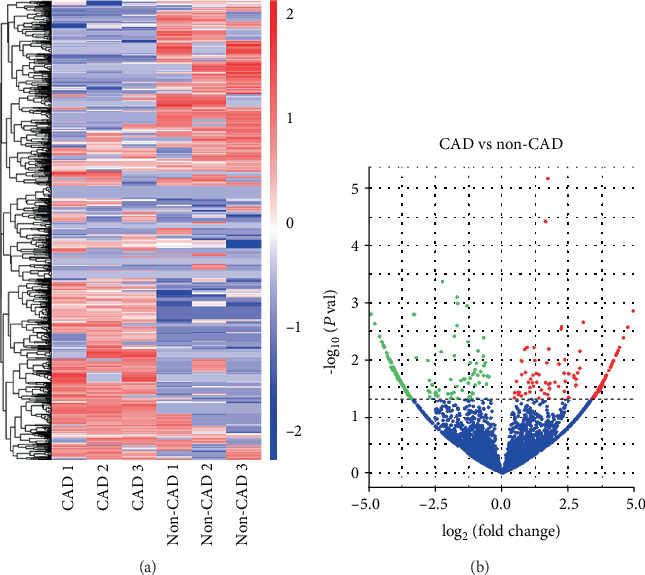
Differentially expressed exosomal circRNAs between CAD patients and non-CAD controls. (a) Hierarchical clustering analysis of exosomal circRNA profile in CAD patients (*n* = 3) and non-CAD controls (*n* = 3). The expression of exosomal circRNAs is hierarchically clustered on the *y*-axis; CAD patients and non-CAD controls are hierarchically clustered on the *x*-axis. Expression values are presented in red and blue to indicate upregulation and downregulation, respectively. (b) Volcano plot: *x*-axis: log_2_(fold change); *y*-axis: -log_10_(*P* value). The red points indicate exosomal circRNAs 1.5-fold upregulated significantly and the green points represent exosomal circRNAs 1.5-fold downregulated with statistical significance.

To identify alterations in circRNA expression in circulating exosomes between CAD patients and non-CAD controls, we first performed circRNA screening. Specifically, the circRNA profiles in isolated circulating exosomes from CAD patients (*n* = 3) and non-CAD controls (*n* = 3) were assessed using an Illumina HiSeq™ 2500. As illustrated in the hierarchical clustering analysis (Figure 1(a)), circRNA expression profiles in CAD patients were distinctly different from those in non-CAD controls. The volcano plot showed the statistical significance of differentially expressed circRNAs between CAD patients and non-CAD controls and identified 355 circRNAs whose levels changed substantially (fold change > 1.5), including 164 upregulated and 191 downregulated circRNAs (Figure 1(b)). We then verified the expression of hsa_circ_0005540, hsa_circ_0000676, and hsa_circ_0007385, which were differentially expressed in the profile phase (fold change > 4, *P* < 0.05).

### 3.2. Internal Validation of Differentially Expressed Exosomal CircRNAs

**Figure 2:**
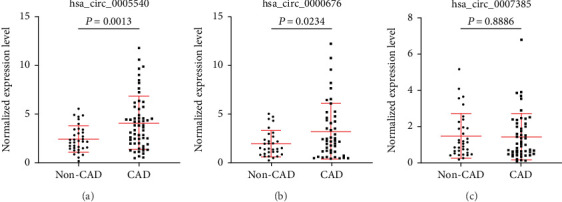
Expression levels of exosomal circRNAs were quantified by qPCR in the internal validation cohort. (a–c) Expression levels of exosomal circRNAs: (a) hsa_circ_0005540, (b) hsa_circ_0000676, and (c) hsa_circ_0007385.

To technically and biologically validate these exosomal circRNAs from the screening stage, we performed quantitative real-time RT-PCR measurements in 58 CAD patients and 35 non-CAD controls, referred to as internal validation cohort. The results showed that hsa_circ_0005540 and hsa_circ_0000676 were significantly increased in CAD patients as compared with non-CAD controls (*P* < 0.05, Figures 2(a) and 2(b)). However, there was no significant difference for hsa_circ_0007385 between CAD patients and non-CAD controls (*P* > 0.05, Figure 2(c)).

### 3.3. External Validation of Differentially Expressed Exosomal CircRNAs

**Figure 3:**
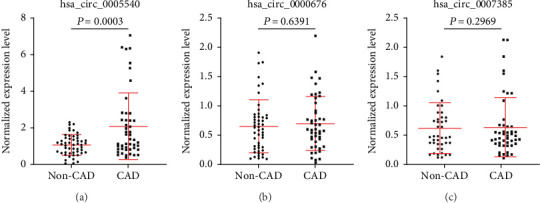
Expression levels of exosomal circRNAs were quantified by qPCR in the external validation cohort. (a–c) Expression levels of exosomal circRNAs: (a) hsa_circ_0005540, (b) hsa_circ_0000676, and (c) hsa_circ_0007385.

To further explore the applicability of these exosomal circRNAs as potential diagnostic biomarkers for CAD, we tested these circRNAs in an external validation cohort consisting of 47 CAD patients and 51 non-CAD controls. As shown in Figure 3(a), only hsa_circ_0005540 was significantly increased in patients with CAD as compared with non-CAD controls (*P* < 0.001), whereas there was no significant difference for hsa_circ_0000676 or hsa_circ_0007385 between the two groups (Figures 3(b) and 3(c)).

### 3.4. Exosomal hsa_circ_0005540 Combined with FHS Risk Factors

**Figure 4:**
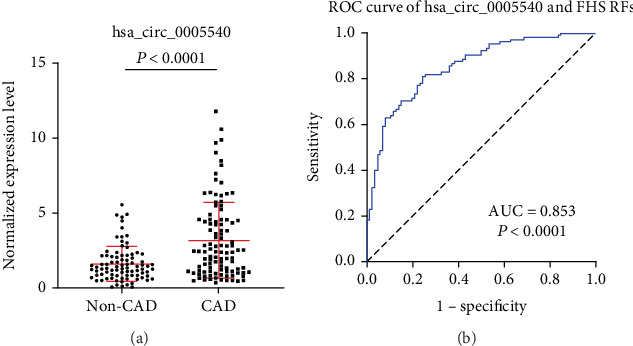
Expression level and ROC curve analysis of exosomal hsa_circ_0005540 in two-center validation cohorts. (a) Expression level of hsa_circ_0005540. (b) ROC curve analysis of exosomal hsa_circ_0005540 combined with FHS RFs for diagnosis of CAD.

As the above results were obtained in two different cohorts, we further assessed hsa_circ_0005540 as a biomarker for CAD in all individuals, including 105 CAD patients and 86 non-CAD controls. Levels of hsa_circ_0005540 were significantly increased in CAD patients as compared with the non-CAD controls (*P* < 0.0001, Figure 4(a)). Then, we examined the correlation between hsa_circ_0005540 levels and the FHS risk factors by Pearson's correlation test. Our data revealed that hsa_circ_0005540 were weakly correlated with the FHS risk factors (*r* = 0.212, *P* < 0.01, Supplementary Figure S1). After adjustment for risk factors, hsa_circ_0005540 showed a high discriminatory power for CAD in ROC analyses (AUC = 0.853; 95%CI = 0.799-0.906, *P* < 0.0001, Figure 4(b)), with the sensitivity of 0.810 and the specificity of 0.765. These results imply that the exosomal hsa_circ_0005540 could serve as a potential diagnostic biomarker for CAD.

## 4. Discussion

Currently, CAD can be diagnosed by either noninvasive or invasive methods, but each has its limitations. Therefore, a highly sensitive and specific biomarker is urgently needed to facilitate the diagnosis of CAD. In this study, we first explored the differentially expressed exosomal circRNAs in CAD patients and non-CAD controls, and potential circRNAs were selected for verification. Our results from clinical samples demonstrated that the levels of exosomal hsa_circ_0005540 in plasma from patients with CAD were significantly elevated compared with those non-CAD controls. The present work has led us to conclude that exosomal circRNAs can be clinically practicable biomarkers for CAD diagnosis.

To date, clinical studies of circRNAs expression in CAD are limited and have produced varying results. Several cross-sectional studies have reported differences in some circRNAs among patients with CAD compared to controls [32–34]. A study from Zhao et al. was the first to investigate the circRNA profile in the peripheral blood of CAD patients and determine its correlation with the severity of CAD, which suggests that hsa_circ_0124644 might be a sensitive and specific biomarker for diagnosing CAD [34]. Another study offered a transcriptome-wide overview of aberrantly expressed circRNAs in CAD patients and identified hsa_circ_0001879 and hsa_circ_0004104 as novel circRNA biomarkers to diagnose CAD [33]. However, no exosomal circRNAs have been reported to be associated with CAD. The aim of this study was to investigate the potential of exosomal circRNAs in plasma as diagnostic biomarkers for CAD. Our results suggest that plasma exosomal hsa_circ_0005540 can be used as a promising diagnostic biomarker for CAD.

hsa_circ_0005540 is located at chr5:94204037-94248681, and the name of its source gene is *MCTP1*, which encodes multiple C2 domain transmembrane protein 1. There is currently no definitive evidence demonstrating the biological function of hsa_circ_0005540. CircRNAs can regulate gene expression by acting as miRNA sponges. There are some binding sites for miR-221 and miR-145 in hsa_circ_0005540, and these miRNAs were associated with CAD [35–38]. Moreover, miR-221 was closely related to endothelial cell survival, migration, and capillary tube formation [35]. miR-145 was explored for its potential as a powerful biomarker for diagnosis of CAD and had been shown to be associated with the severity of CAD [36, 38]. Therefore, we speculate that hsa_circ_0005540 is involved in the progression of CAD.

In addition to CAG, many methods are currently used to diagnose CAD, including routine ECG, Holter monitoring, TET, and CTA. The sensitivities of these methods have been shown to be 0.29, 0.65, 0.79, and 0.92, respectively [39–42]. The specificities are 0.67, 0.90, 0.80, and 0.75, respectively [39–42]. In our study, we found that the sensitivity and specificity of exosomal hsa_circ_0005540 combined with FHS risk factors were 0.810 and 0.765, respectively. Based on these comparisons, the diagnostic value of exosomal hsa_circ_0005540 combined with FHS risk factors was greater than that of routine ECG and TET, which was approximately equal to Holter monitoring. When considering the cost and convenience of diagnostic methods, exosomal hsa_circ_0005540 might ameliorate the diagnosis of CAD.

It should be pointed out that there were some limitations in our study. Although 2 independent cohorts for validation had been performed in our study, a larger clinical population is warranted to further validate the performance of the association of exosomal hsa_circ_0005540 as a potential blood-based signature in CAD. Furthermore, since the levels of exosomal circRNAs may be affected by multiple parameters such as the change in expression in the tissue and the release of exosomes by the cells into the circulation, further studies are necessary to explore the mechanisms underlying the dysregulation and the putative impact of the changes of exosomal hsa_circ_0005540 level in the pathophysiology.

## 5. Conclusion

In conclusion, our study reported a novel signature of exosomal hsa_circ_0005540, allowing to distinguish patients with CAD from non-CAD controls, and provided first insights into the levels of exosomal circRNAs in patients with CAD that could be translated into noninvasive blood-based biomarker.

## Figures and Tables

**Table 1:** Characteristics of the study populations.

Characteristics	Profiling phase		Internal validation phase		External validation phase	
	Non-CAD (*n* = 3)	CAD (*n* = 3)	Non-CAD (*n* = 35)	CAD (*n* = 58)	Non-CAD (*n* = 51)	CAD (*n* = 47)
Age (years)	55.67 ± 12.06	67.33 ± 4.51	59.69 ± 10.92	61.88 ± 10.21	61.43 ± 13.50	67.87 ± 9.23
Sex (male)	2 (66.67%)	1 (33.33%)	13 (37.14%)	36 (62.06%)	19 (37.25%)	35 (74.47%)
Smoking	0 (0%)	1 (33.33%)	5 (14.28%)	26 (44.83%)	0 (0%)	8 (17.02%)
Hypertension	1 (33.33%)	3 (100.00%)	12 (34.29%)	37 (63.79%)	9 (17.65%)	16 (34.04%)
Diabetes	0 (0%)	2 (66.67%)	1 (2.86%)	18 (31.03%)	5 (9.80%)	16 (34.04%)
Systolic BP (mmHg)	108.00 ± 6.00	139.30 ± 10.07	120.60 ± 24.99	131.10 ± 21.79	122.4 ± 17.78	142.30 ± 21.17
FPG (mM)	4.63 ± 0.46	6.69 ± 2.10	4.77 ± 0.48	5.71 ± 2.09	5.24 ± 1.11	5.92 ± 2.27
TC (mM)	2.81 ± 0.19	4.25 ± 0.69	4.85 ± 1.13	4.62 ± 1.00	5.07 ± 0.82	4.80 ± 1.66
HDL (mM)	1.41 ± 0.23	0.85 ± 0.22	1.22 ± 0.26	1.06 ± 0.30	1.48 ± 0.33	1.22 ± 0.35
Coronary artery disease						
1 vessel		1 (33.33%)		25 (43.10%)		17 (36.17%)
2 vessels		1 (33.33%)		24 (41.38%)		19 (40.43%)
3 vessels		1 (33.33%)		9 (15.52%)		11 (23.40%)

Data are summarized by either mean standard ± deviation. Abbreviations: BP: blood pressure; FPG: fasting plasma glucose; TC: total cholesterol; HDL: high-density lipoprotein.

## Data Availability

Raw and normalized data files for the RNA-seq analysis have been deposited in the NCBI Gene Expression Omnibus under accession number GSE152498. You may view the GSE152498 study at https://www.ncbi.nlm.nih.gov/geo/query/acc.cgi?acc=GSE152498.

## Supplementary Materials

Supplementary Materials Table S1: sequences of all real-time PCR primers. Figure S1: the Pearson correlation coefficient between hsa_circ_0005540 and FHS risk factors.
