# Supplementary material for: Plasma-Derived Exosomal Circular RNA hsa_circ_0005540 as a Novel Diagnostic Biomarker for Coronary Artery Disease
Source: Dis Markers. 2020 Jun 30;2020:3178642. doi: 10.1155/2020/3178642 (PMC7346252; doi:10.1155/2020/3178642)
Supplement: Supplementary Materials — Table S1: sequences of all real-time PCR primers. Figure S1: the Pearson correlation coefficient between hsa_circ_0005540 and FHS risk factors. [file 3178642.f1.docx]

**Supplementary Material**

TABLE S1: Sequences of real-time PCR primers.

| Primer Name | Sequence (5’-3’) |
| --- | --- |
| ACTIN-F | AAGATGACCCAGATCATGTTTGAG |
| ACTIN-R | GCAGCTCGTAGCTCTTCTCCAG |
| hsa_circ_0005540-F | GAGGAAAACAGACTCTCTAAACAGC |
| hsa_circ_0005540-R | TGTGTAGGTCTGATAGGCGTAAA |
| hsa_circ_0007385-F | GATTTACGGAGAACTGTGGAAGAC |
| hsa_circ_0007385-R | AAGCATGGGCAGCACAAGA |
| hsa_circ_0000676-F | CGTCTACTTTTCCCTCTTACTCATT |
| hsa_circ_0000676-R | AGGACCGTGTTCTGAAAGCA |


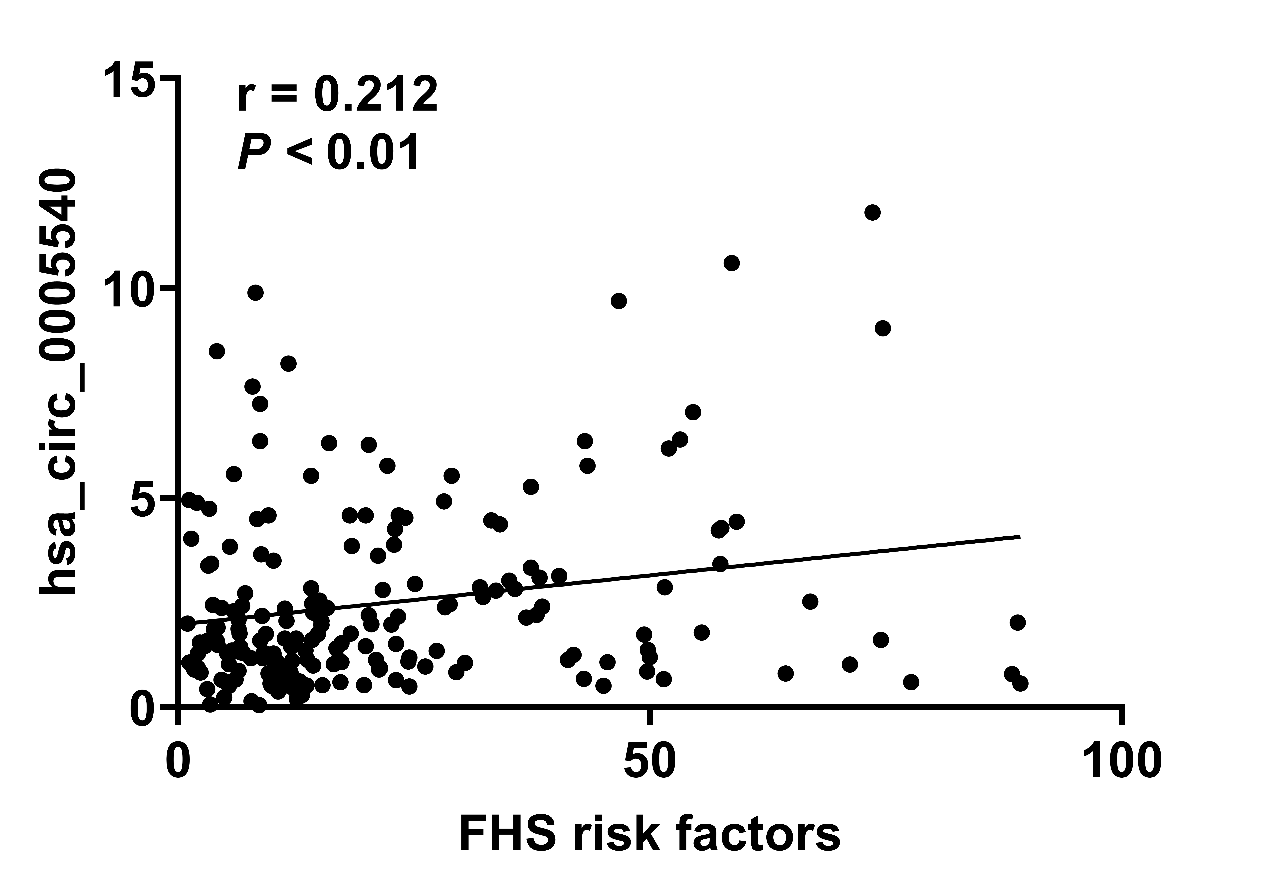


FIGURE S1: Pearson correlation coefficient between hsa_circ_0005540 and FHS risk factors.
